# Effect of acute pancreatitis on the risk of developing osteoporosis: A nationwide cohort study

**DOI:** 10.1371/journal.pone.0179358

**Published:** 2017-06-12

**Authors:** Shih-Yi Lin, Wu-Huei Hsu, Cheng-Chieh Lin, Cheng-Li Lin, Chung-Hao Tsai, Chia-Hung Kao

**Affiliations:** 1 Graduate Institute of Clinical Medical Science, College of Medicine, China Medical University, Taichung, Taiwan; 2 Division of Nephrology and Kidney Institute, China Medical University Hospital, Taichung, Taiwan; 3 Department of Chest, China Medical University Hospital, Taichung, Taiwan; 4 Department of Family Medicine, China Medical University Hospital, Taichung, Taiwan; 5 Management Office for Health Data, China Medical University Hospital, Taichung, Taiwan; 6 College of Medicine, China Medical University, Taichung, Taiwan; 7 Department of Orthopedics, China Medical University Hospital, Taichung, Taiwan; 8 Department of Nuclear Medicine and PET Center, China Medical University Hospital, Taichung, Taiwan; 9 Department of Bioinformatics and Medical Engineering, Asia University, Taichung, Taiwan; Universitat de Valencia, SPAIN

## Abstract

**Purpose:**

Chronic exocrine pancreatic insufficiency can lead to osteoporosis. However, the incidence and risk of osteoporosis after acute inflammation of pancreas remained known. Thus, we conducted a population-based cohort study to clarify the association between acute pancreatitis (AP) and osteoporosis.

**Methods:**

Patients newly diagnosed with AP with index date between 2000 and 2011 were identified from the National Health Insurance Research Database. Osteoporosis were defined according to the International Classification of Diseases, Ninth Revision, Clinical Modification codes. We applied age-, sex-, and comorbidities-adjusted variable Cox proportional hazard models for assessing the association between AP and osteoporosis. Moreover, these models were used to adjust for the influences of patient characteristics and comorbidities.

**Results:**

In this study, 4,016 patients were included in the AP cohort (males, 67.9%; mean age, 51.8 years) and 4,016 matched controls in the non-AP cohort. After a mean follow-up period of 4.97 and 5.21 years in the AP and non-AP cohorts, respectively, the incidence of osteoporosis was 8.22 per 1000 person-years in the AP cohort. The AP cohort had a higher risk [adjusted hazard ratio (aHR) = 1.27, 95% confidence interval (CI) = 1.02–1.58] of osteoporosis than did the non-AP cohort. The risk of osteoporosis was highest in the female patients of the AP cohort (aHR = 2.26, 95% CI = 1.85–2.76) and patients aged 50–64 years (aHR = 4.14, 95% CI = 3.13–5.47).

**Conclusion:**

AP patients are at a risk of osteoporosis, especially female gender and age 50–64 years. Those with > 3 episodes of AP had highest significant risk of developing osteoporosis.

## Introduction

Osteoporosis, characterized by reduced bone mineral density and distorted bone microstructure, markedly increases the risk of fractures [1, 2]. Osteoporotic fractures typically develop after low-energy traumas to the hip, spine, proximal humerus, and forearm[3]. Furthermore, achieving full recovery of osteoporotic fractures is difficult. Patients with osteoporotic fractures, particularly elderly patients, often lose activities of daily living and independence. Approximately 20% of the patients with osteoporotic fractures required long-term nursing care, and approximately 20% died one year later [4]. Thus, early detection and prevention of osteoporosis is a critical step for reducing the national economic and care burden. Osteoporosis is defined as bone mineral density measured by dual energy X-ray absorptiometry of the lumbar spine, T-score ≤ −2.5 SD, or presence of fragility fractures [5]. Pharmacological prevention and intervention of osteoporosis includes provision of calcium supplement (1,200 mg/day), vitamin D (800–1,000 IU/day), bisphosphonate, raloxifene, denosumab, teriparatide, and calcitonin or hormone therapy [1]. Nonpharmacological interventions for osteoporosis include moderate exercise, adequate sunlight exposure, smoking cessation, and most importantly, identification of risk factors [4].

Clinical risk factors for osteoporosis, including postmenopausal complications, long-term steroid use, alcoholism, rheumatological disorders, renal failures, gastrointestinal disorders (gastric bypass, biliary cirrhosis, and pancreatic insufficiency), and malnutrition (anorexia nervosa, vitamin A excess, and vitamin D deficiency) have been identified [6, 7]. Chronic pancreatitis (CP) has been reported to be associated with osteoporosis and decreased bone mineral density [1, 7]. It has been reported that even one episode of AP may progress to CP-like lesions with calcifications [8]. However, whether acute inflammation of the pancreas is associated with osteoporosis still remains unknown. Therefore, we used the National Health Insurance Registration Database (NHIRD) for investigating the association between acute pancreatitis (AP) and osteoporosis.

## Methods

### Data source

All data used in this study were retrieved from the Longitudinal Health Insurance Database 2000 (LHID2000)—a subset of the NHIRD. The LHID2000 contains longitudinal data (1996–2011) on medical claims for 1 million individuals randomly selected from the 2000 Registry of Beneficiaries (approximately 24 million) of Taiwan’s National Health Insurance (NHI) program [9]. Data from the LHID2000 have been used successfully in numerous epidemiological studies [9–11].

### Ethics statement

The NHIRD encrypts patient personal information to protect privacy and provides researchers with anonymous identification numbers associated with relevant claims information, including sex, date of birth, medical services received, and prescriptions. Therefore, patient consent is not required to access the NHIRD. This study was approved to fulfill the condition for exemption by the Institutional Review Board (IRB) of China Medical University (CMUH104-REC2-115-CR1). The IRB also specifically waived the consent requirement.

### Participants

The AP cohort included patients newly diagnosed with acute pancreatitis [the International Classification of Diseases, Ninth Revision, Clinical Modification (ICD-9-CM) 577.0] from 2000 to 2010; In this study, the criteria of acute pancreatitis (AP) cohort are defined as who had the main diagnosis of AP (ICD-9 577.0) in inpatient benefit claims of NHIRD. Each diagnosis of AP is based on clinicians’ decisions of typical abdominal pain, elevating levels of amylase and lipase, and imaging findings, although the laboratory information is unavailable in this NHIRD releasing for research purpose. Professionals in NHI periodically supervise and review these diagnosis and laboratory information to ensure the diagnosis and avoid wastes of medical resources. Thus, in NHIRD, the diagnosis of AP is according to the well-established and updated international criteria. The accuracy of AP diagnosis (ICD-9-CM-577.0) in NHIRD has been validated and been used to conduct several NHIRD-based studies [12–14]. The diagnosis of AP is accurate in this study. The first diagnosis date of AP was considered the index date. Patients with a history of CP (ICD-9-CM 577.0), those with osteoporosis (ICD-9-CM 733.0, 733.1) before index date, or those aged <20 years were excluded. The non-AP cohort was also identified during 2000–2010, with an exclusion criteria similar to that of the AP cohort. A propensity score matching (1:1) was performed in the AP and non-AP cohorts. The propensity score was calculated using logistic regression analysis for estimating the probability of osteoporosis, given baseline variables, including age, hypertension (ICD-9-CM 401–405), diabetes mellitus (ICD-9-CM 250), hyperlipidemia (ICD-9-CM 272), stroke (ICD-9-CM 430–438), chronic obstructive pulmonary disease (COPD; ICD-9-CM 491, 492, and 496), cirrhosis (ICD-9-CM 571.2, 571.5, and 571.6), chronic kidney disease (ICD-9-CM 580–589), cancer (ICD-9-CM 140–208), folate deficiency (ICD-9-CM 281), depression (ICD-9-CM 296.2, 296.3, 300.4, and 311), hyperthyroidism (ICD-9-CM 242), hypothyroidism (ICD-9-CM 244), fibromyalgia (ICD-9-CM 729.0 and 729.1), coronary artery disease (ICD-9-CM 410–414), alcohol-related diseases (ICD-9-CM 291, 303, 305.0, 571.0–571.3, 790.3, and V11.3), biliary stone (ICD-9-CM 574), asthma (ICD-9-CM 493), hypercalcemia (ICD-9-CM 275.42), and hyperparathyroidism (ICD-9-CM 252), and medication of steroid. Severe acute pancreatitis is considered here as acute pancreatitis coexisting with acute kidney injury (ICD-9-CM 584) or multiple organ failure (ICD-9-CM 995.92) or acute liver failure (ICD-9-CM 570).

### Outcome measurements

Both AP and non-AP cohorts were followed until osteoporosis diagnosis, death, withdrawal from the NHI program, or the end of 2011, whichever occurred first.

### Statistical analysis

The differences in the demographic characteristics of the AP and non-AP cohorts were evaluated using the chi-squared and Student’s t tests for categorical and continuous variables, respectively. The different risk factors for osteoporosis were identified and stratified by age, sex, and comorbidities in both the cohorts. Univariable and multivariable competing-risks regression models were used for estimating the subhazard ratios (SHRs) with 95% confidence intervals (CIs) for osteoporosis associated with AP. The multivariable models were adjusted for age, sex, and comorbidities, namely hypertension, folate deficiency, depression, fibromyalgia, and coronary artery disease. We estimated the cohort-specific probability for osteoporosis by directly adjusting the survival function for age, sex, and comorbidities in the multivariable competing-risks Cox model. SAS version 9.4 for Windows (SAS Institute, Cary, NC, USA) was used for conducting statistical analyses. P < .05 was considered significant for a 2-tailed test.

## Results

As demonstrated in Table 1, both cohorts exhibited similar age, sex, and comorbidity distributions, and the patients were predominantly male (68.1%). The mean ages in the AP and non-AP cohorts were 51.8 (SD = 17.2) and 52.5 (SD = 17.0) years, respectively. In the AP and non-AP cohorts, hypertension (39.7% vs 38.3%, respectively) was the predominant comorbidity followed by cirrhosis (36.9% vs 34.7%, respectively), hyperlipidemia (27.9% vs 27.4%, respectively), and coronary artery disease (19.1% vs 19.3%, respectively). The mean follow-up periods of AP and non-AP cohorts were 4.97 and 5.21 years, respectively. In AP cohort, 17 cases are defined as severe acute pancreatitis. Among these 17 severe acute pancreatitis patients, none of them develop osteoporosis.

**Table 1:** Characteristics between subjects with and without acute pancreatitis.

	Acute pancreatitis				
	Yes		No		
	(N = 4016)		(N = 4016)		
	n	%	n	%	*p*-value
**Age, year Mean (SD)**^#^	51.8	17.2	52.5	17.0	0.08
**Sex**					0.79
Female	1291	32.2	1280	31.9	
Male	2725	67.9	2736	68.1	
**Comorbidity**					
Hypertension	1537	38.3	1594	39.7	0.19
Diabetes mellitus	695	17.3	714	17.8	0.58
Hyperlipidemia	1101	27.4	1120	27.9	0.64
Stroke	305	7.59	337	8.39	0.19
COPD	569	14.2	606	15.1	0.24
Cirrhosis	1393	34.7	1483	36.9	0.04
CKD	183	4.56	179	4.46	0.83
Cancer	198	4.93	198	4.93	0.99
Folate deficiency	25	0.62	24	0.60	0.89
Depression	281	7.00	267	6.65	0.54
Hyperthyroidism	48	1.20	46	1.15	0.84
Hypothyroidism	22	0.55	23	0.57	0.88
Fibromyalgia	714	17.8	724	18.0	0.77
Coronary artery disease	773	19.3	765	19.1	0.82
Alcohol-related diseases	590	14.7	639	15.9	0.13
Biliary stone	401	9.99	453	11.3	0.06
Asthma	315	7.84	329	8.19	0.57
Hypercalcemia	3	0.07	1	0.02	0.32
Hyperparathyroidism	12	0.30	9	0.22	0.51
Coexisting complications					
Sepsis	39	0.97	111	2.76	0.001
Biliary tract disease	47	1.17	73	1.82	0.02
**Medication**					
Steroid	347	8.64	332	8.27	0.55

Chi-square test;

^#^ Student’s *t*-test;

**Fig 1:**
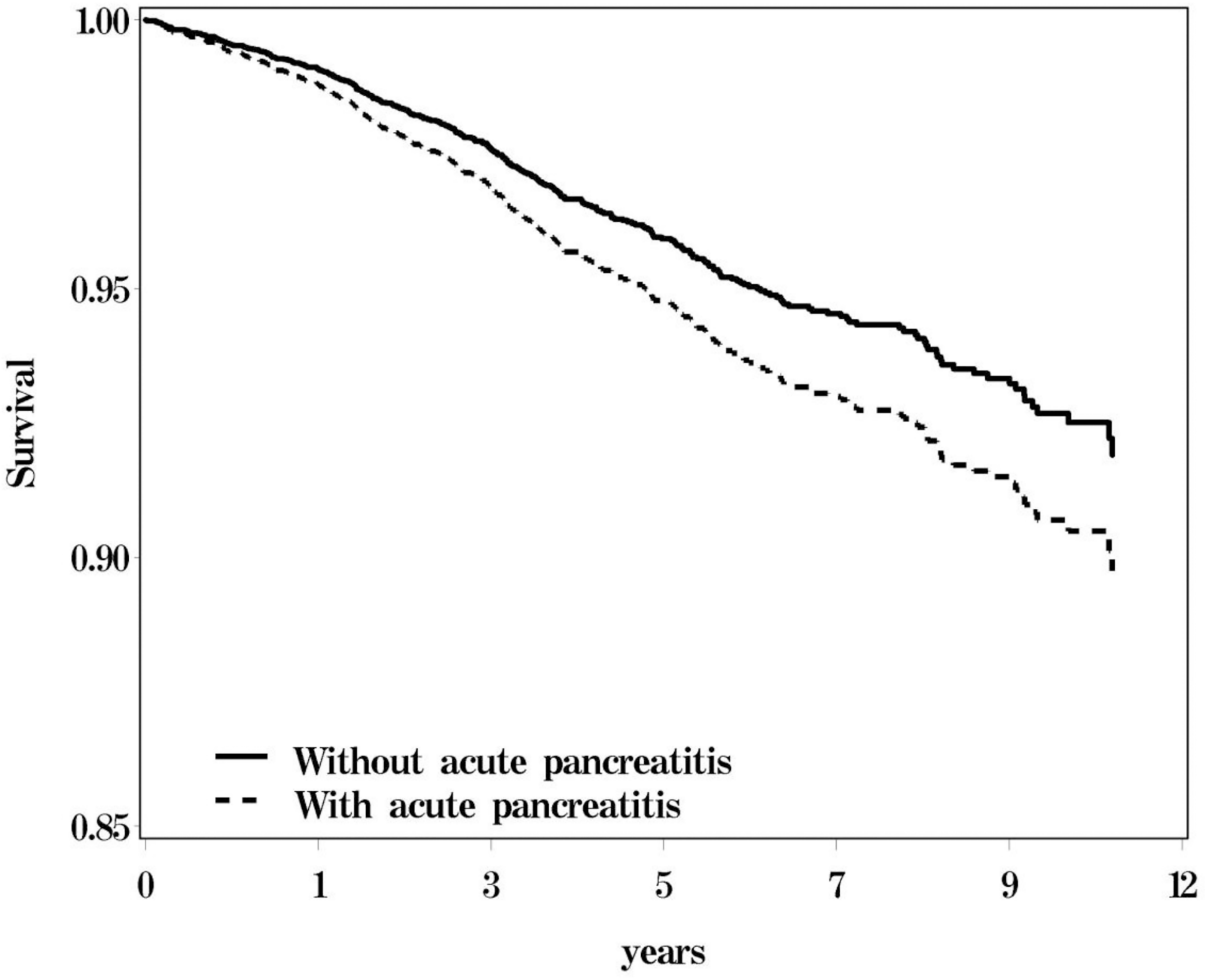
Probability for osteoporosis between subjects with and without acute pancreatitis.

Fig 1 presents the 12-year probability for osteoporosis associated with AP adjusted for age, sex, and comorbidities. The patients with cholangitis and those without AP exhibited significant differences in their probability values for osteoporosis. The overall incidence of osteoporosis (per 1000 person-years) in the AP and non-AP cohorts was 8.22 and 7.03, respectively (crude SHR = 1.35, 95% CI = 1.09–1.68; Table 2). After adjustment for all confounding factors in the competing-risks regression model, the risk of osteoporosis remained significantly increased in the presence of AP (adjusted SHR = 1.27, 95% CI = 1.02–1.58). Compared with patients aged ≤49 years, those aged 50–64 years had a 4.14-fold risk (95% CI = 3.13–5.47) of osteoporosis, and patients aged ≥65 years had a 2.01-fold risk (95% CI = 1.53–2.65) of osteoporosis. The risk of osteoporosis was 2.26-fold higher in women than in men (95% CI = 1.85–2.76).

**Table 2:** The incidences and risk factors for osteoporosis.

Variable	Event	PY	Rate^#^	Crude SHR (95% CI)	Adjusted SHR^&^ (95% CI)
**Acute pancreatitis**					
No	147	20918	7.03	1.00	1.00
Yes	164	19961	8.22	1.35(1.09, 1.68)**	1.27(1.02, 1.58)*
**Age, year**					
20–49	43	23449	1.83	1.00	1.00
50–64	94	9557	9.84	3.14(2.44, 4.05)***	4.14(3.13, 5.47)***
≥ 65	174	7874	22.1	6.45(5.22, 8.00)***	2.01(1.53, 2.65)***
**Sex**					
Female	202	12841	15.7	2.82(2.27, 3.50)***	2.26(1.85, 2.76)***
Male	109	28039	3.89	1.00	1.00
**Comorbidity**					
**Hypertension**					
No	115	27130	4.24	1.00	1.00
Yes	196	13750	14.3	1.47(1.14, 1.89)**	1.05(0.81, 1.35)
**Diabetes mellitus**					
No	238	34740	6.85	1.00	1.00
Yes	73	6140	11.9	1.09(0.83, 1.42)	-
**Hyperlipidemia**					
No	210	30211	6.95	1.00	1.00
Yes	101	10669	9.47	1.01(0.80, 1.27)	-
**Stroke**					
No	227	38802	7.14	1.00	1.00
Yes	34	2078	16.4	0.88(0.60, 1.29)	-
**COPD**					
No	239	36099	6.62	1.00	1.00
Yes	72	4781	15.1	1.29(0.99, 1.67)	-
**Cirrhosis**					
No	175	26441	6.62	1.00	1.00
Yes	136	14439	9.42	1.15(0.93, 1.43)	-
**CKD**					
No	294	39668	7.41	1.00	1.00
Yes	17	1212	14.0	1.07(0.67, 1.70)	-
**Cancer**					
No	297	39794	7.46	1.00	1.00
Yes	14	1086	12.9	0.89(0.52, 1.53)	-
**Folate deficiency**					
No	307	40734	7.54	1.00	1.00
Yes	4	146	27.4	2.45(1.01, 5.94)*	1.84(0.68, 4.93)
**Depression**					
No	281	38557	7.29	1.00	1.00
Yes	30	2323	12.9	1.53(1.07, 2.20)*	1.08(0.74, 1.58)
**Hyperthyroidism**					
No	306	40490	7.56	1.00	1.00
Yes	5	390	12.8	1.02(0.38, 2.74)	-
**Hypothyroidism**					
No	308	40725	7.56	1.00	1.00
Yes	3	155	19.3	2.37(0.88, 6.35)	-
**Fibromyalgia**					
No	234	34620	6.76	1.00	1.00
Yes	77	6260	12.3	1.58(1.24, 2.02)***	1.13(0.87, 1.46)
**Coronary artery disease**					
No	207	34367	6.02	1.00	1.00
Yes	104	6513	16.0	1.32(1.03, 1.68)*	0.98(0.75, 1.27)
**Alcohol-related diseases**					
No	280	35531	7.88	1.00	1.00
Yes	31	5348	5.80	1.03(0.73, 1.46)	-
**Biliary stone**					
No	255	36683	6.95	1.00	1.00
Yes	56	4196	13.3	1.09(0.81, 1.47)	-
**Asthma**					
No	270	38108	7.09	1.00	1.00
Yes	41	2772	14.8	1.36(0.99, 1.87)	-
**Hypercalcemia**					
No	311	40870	7.61	1.00	1.00
Yes	0	10	0.00	-	-
**Hyperparathyroidism**					
No	311	40799	7.62	1.00	1.00
Yes	0	81	0.00	-	-
Coexisting complications					
Sepsis					
No	303	40353	7.51	1.00	1.00
Yes	8	527	15.2	1.01(0.50, 2.03)	-
Yes	7	595	11.8	0.81(0.39, 1.70)	-
**Medication**					
**Steroid**					
No	206	33516	6.15	1.00	1.00
Yes	105	7364	14.3	1.42(1.02, 1.99)*	1.29(0.92, 1.81)

CI, confidence interval; SHR, subhazard ratio; PY, person-years;

Rate^#^, incidence rate, per 1,000 person-years;

^&^Multivariable analysis including age, sex, hypertension, folate deficiency, depression, fibromyalgia, and coronary artery disease, and medications of steroid;

*p<0.05

**p<0.01

***p<0.001

Table 3 presents the comparison of the SHRs for osteoporosis obtained from the competing-risks regression models for the AP and non-AP cohorts stratified by age, sex, and comorbidities. The incidence of osteoporosis increased with age in both the cohorts. The age-specific relative risk of osteoporosis in the AP cohort was higher than that in the non-AP cohort for patients aged 50–64 years (adjusted SHR = 1.99, 95% CI = 1.33–2.97). The sex-specific relative risk of osteoporosis in the AP cohort was higher than that in the non-AP cohort for women (adjusted SHR = 1.43, 95% CI = 1.10–1.85). Table 4 indicates that compared with patients without AP, patients who experienced >3 episodes of AP had a 4.76-fold risk of osteoporosis.

**Table 3:** Incidences and subhazard ratios of osteoporosis between subjects with and without acute pancreatitis.

	Acute pancreatitis							
	Yes			No				
Variable	Event	PY	Rate^#^	Event	PY	Rate^#^	Crude SHR (95% CI)	Adjusted SHR^&^ (95% CI)
**Age, year**								
20–49	27	11954	2.26	16	11496	1.39	1.59(0.96, 2.64)	1.53(0.87, 2.68)
50–64	53	4457	11.9	41	5100	8.04	1.72(1.20, 2.46)**	1.99(1.33, 2.97)***
≥ 65	84	3551	23.7	90	4323	20.8	1.18(0.91, 1.53)	1.19(0.90, 1.59)
**Sex**								
Female	113	6162	18.3	89	6679	13.3	1.47(1.12, 1.93)**	1.43(1.10, 1.85)**
Male	51	13799	3.70	58	14239	4.07	0.82(0.58, 1.17)	0.92(0.63, 1.35)
**Comorbidity**^§^								
No	16	4976	3.22	13	6408	2.03	1.70(0.85, 3.41)	2.08(1.01, 4.26)*
Yes	148	14986	9.88	134	14510	9.23	1.08(0.86, 1.34)	1.19(0.95, 1.49)
Steroid								
No	139	18795	7.39	132	19777	6.67	1.08(0.86, 1.35)	1.22(0.97, 1.53)
Yes	25	1163	21.5	15	1141	13.2	1.66(0.91, 3.04)	1.92(1.04, 3.53)*

CI, confidence interval; SHR, subhazard ratio; PY, person-years;

Rate^#^, incidence rate, per 1,000 person-years;

^&^Multivariable analysis including age, sex, hypertension, folate deficiency, depression, fibromyalgia, and coronary artery disease, and medications of steroid;

^§^Subjects with any comorbidity of hypertension, diabetes mellitus, hyperlipidemia, stroke, COPD, cirrhosis, CKD, cancer, folate deficiency, depression, hyperthyroidism, hypothyroidism, fibromyalgia, coronary artery disease, alcohol-related diseases, biliary stone, asthma, hypercalcemia, and hyperparathyroidism were classified into the comorbidity group;

*p<0.05

**p<0.01

***P<0.001

**Table 4:** The risk of osteoporosis among acute pancreatitis patients receiving different frequency for medical visits in Cox proportional hazard regression.

Frequency for medical visit, per 1 years	Event	PY	Rate^#^	Crude SHR (95% CI)	Adjusted SHR^&^ (95% CI)
**None-acute pancreatitis**	147	20918	7.03	1.00	1.00
**Acute pancreatitis**					
≦3	143	19264	7.42	1.02(0.82, 1.27)	1.19(0.95, 1.50)
> 3	21	697	30.1	3.84(2.43, 6.05)***	4.76(2.99, 7.59)***
p for trend					<0.0001
Frequency for medical visits				1.01(1.01, 1.02)***	1.01(1.01, 1.02)***

CI, confidence interval; PY, person-years; SHR, subhazard ratio;

Rate^#^, incidence rate, per 1,000 person-years;

^&^Multivariable analysis including age, sex, hypertension, folate deficiency, depression, fibromyalgia, and coronary artery disease, and medications of steroid;

***P<0.001

## Discussion

To our knowledge, this study is the first reporting the association of AP with osteoporosis, especially with the significant risk factors, including female gender and age > 65 years. In the present study, we used propensity score weighting to adjust for a wide range of confounding factors of osteoporosis. Our analysis revealed that compared with the non-AP cohort, the AP cohort had a 1.27-fold risk of osteoporosis. The risk of osteoporosis was 4.71 in patients who experienced >3 episodes of AP. The presumed association between AP and osteoporosis, was confirmed by this study. We found that AP has remote lasting effects on bone mineral density rather than just a single episode of pancreas inflammation.

We proposed several explanations for our findings that the AP cohort had a higher risk of osteoporosis. First, AP progresses to CP with time. Although the underlying mechanism is unclear, the pancreas developed CP-like lesions with calcifications after an episode of AP [8]. Yadav et al. reported recurrent episodes of AP, smoking, and alcohol as some of the risk factors for progression to CP [15]. Furthermore, Haaber et al. reported an association between CP and decreased bone mass [16]. Our finding that patients who experienced >3 episodes of AP were at a relatively higher risk of osteoporosis was consistent with the aforementioned findings. Episodes of AP would result in progression to CP, causing pancreatic insufficiency [17]. Malabsorption of fat occurs because of a shortage in pancreatic enzymes, resulting in fat-soluble (A, D, E, and K) vitamin deficiencies. Vitamin K participated in the gamma-carboxylation of osteocalcin, protecting against osteoporosis [18]. The chief hormonal form of vitamin D, 1,25(OH)_2_D, stimulates various signaling pathways and calcium transport across the cell membranes [19]. Patients with prolonged vitamin K and vitamin D deficiencies might be predisposed to osteoporosis.

Second, episodes of AP induce systemic inflammation, causing impaired bone metabolism and osteoporosis. A study hypothesized that even a single AP episode can initiate a sentinel of uncontrolled inflammatory processes [20]. High levels of chemokines, such as CCL2/MCP-1, neutrophil chemoattractant, CX3CL1/fractalkine, CXCR2, CCR1, interleukin (IL)-6, IL-8, IL-10, and soluble TNF-gamma, have been reported in AP or CP [21–24]. Duggan et al. reported that patients with CP who developed osteoporosis exhibited high levels of IL-6 and high-sensitivity C-reactive protein [21]. Redlich et al. indicated that chronic inflammation in the entire body could affect bone metabolism and induce bone loss [25]. Several chronic inflammation disorders, such as fibromyalgia [26], COPD [27], and peritonitis [28], have been strongly associated with osteoporosis. To determine the association between AP and osteoporosis, we excluded patients with CP to avoid CP interference. Furthermore, we proposed that single or multiple episodes of AP might induce a cascade of inflammatory processes in the pancreas, exerting systemic effects on bone metabolism and consequently causing osteoporosis. This may be another possible mechanism contributing to the high risk of osteoporosis in patients with AP, apart from the AP-induced pancreatic exocrine insufficiency pathway.

However, our study has a few limitations. First, information on the individual characteristics, such as smoking, alcohol consumption, exercise, and sunlight exposure, that might be risk factors for osteoporosis were unavailable in the NHIRD. For overcoming this limitation, we considered comorbidities, such as COPD and alcohol-related diseases, as alternatives to the smoking and alcohol-drinking habits, respectively. Moreover, Taiwan is a small island located within 22–25° 0' N; therefore, differences because of variable sunlight exposure among individuals would be subtle. Second, detailed data of pancreatic function tests, secretin-enhanced magnetic retrograde cholangiopancreatography, or endoscopic ultrasound for delineating the pancreatic morphology and functional capacity of each individual were not obtained. Therefore, the association between the sequels of AP, i.e. morphologic changes and exocrine functional changes of pancreas, and risk of osteoporosis could not be investigated. Finally, because the NHIRD encrypts patient personal information to protect privacy and provides researchers with anonymous identification numbers, therefore, lack of individual subject’s BMD and Z-score data is the other study limitation.

Our study has several notable advantages. First, the NHIRD has a universal coverage and is a comprehensive longitudinal medical database of insureds. NHIRD covers a highly representative sample of Taiwan’s general population because the reimbursement policy is universal and operated by a single-buyer, the government in Taiwan. All insurance claims should be scrutinized by medical reimbursement specialists and peer review according to the standard diagnosed criteria (such as BMD) in the study. If these doctors or hospitals make wrong diagnoses or coding, they will be punished with a lot of penalties. Therefore, the diagnoses of osteoporosis based on ICD-9 codes in this study were highly reliable. In addition, some related studies with the same diagnosed method and criteria for osteoporosis by ICD-9 coding were already been published [29–33]. Second, our study considered the comorbidities that could be risk factors for osteoporosis and propensity-score matched these comorbidities between the study (AP) and control (non-AP) cohorts. In addition, our study design minimized the potential selection bias and clearly investigated the association between AP and osteoporosis.

Furthermore, our study suggested that AP is associated with an increased risk of osteoporosis. This large nationwide cohort study provided clinical information that may be useful in the primary prevention of osteoporosis in patients with AP.

## Supporting information

S1 Checklist Checklist of items that should be included in reports of observational studies. (DOC)

## Abbreviations

aHR: adjusted hazard ratio
AP: acute pancreatitis
CI: confidence interval
CP: Chronic pancreatitis
ICD-9-CM: International Classification of Diseases, Ninth Revision, Clinical Modification
LHID2000: Longitudinal Health Insurance Database 2000
NHIRD: National Health Insurance Registration Database
